# ﻿Molecular and biometric data on *Carabus (Macrothorax) morbillosus* Fabricius, 1792 (Coleoptera, Carabidae) from Mid Mediterranean areas

**DOI:** 10.3897/zookeys.1127.84920

**Published:** 2022-11-02

**Authors:** Mariastella Colomba, Gabriella Lo Verde, Fabio Liberto, Armando Gregorini, Ignazio Sparacio

**Affiliations:** 1 Department of Biomolecular Sciences, University of Urbino Carlo Bo, Via Maggetti 22, 61029 Urbino (PU), Italy University of Urbino Carlo Bo Urbino Italy; 2 Dipartimento di Scienze Agrarie, Alimentari e Forestali (SAAF), Università degli Studi di Palermo, Viale delle Scienze edificio 5, 90128 Palermo, Italy Università degli Studi di Palermo Palermo Italy; 3 Via del Giubileo Magno 93, 90015 Cefalù (PA), Italy Unaffiliated Cefalù Italy; 4 Via Principe di Paternò 3, 90144 Palermo, Italy Unaffiliated Palermo Italy

**Keywords:** Bayesian analysis, biogeography, carabids, COI, elytra, morphometric analysis, pronotum, taxonomy

## Abstract

The present study was carried out using molecular and biometric data of Carabus (Macrothorax) morbillosus from mid-Mediterranean areas to determine additional information on basal relationships among its representative subspecies. To this aim, two different kinds of approach were employed, including a morphometric analysis of four morphological parameters (i.e., elytra length, elytra width, pronotum length, pronotum width) of 128 specimens, and a Bayesian genetic analysis of 44 cytochrome oxidase subunit I (COI) partial sequences (i.e., 38 examined for the first time and six retrieved from GenBank database). Representative populations of C. (M.) morbillosus were sampled in four countries, namely Italy, Malta, Spain, and Tunisia. The present findings support the validity of four C. (M.) morbillosus subspecies, specifically C. (M.) m. alternans, C. (M.) m. bruttianus, C. (M.) m. constantinus, and C. (M.) m. macilentus, and redefine these subspecies’ distributions. Notably, within the C. (M.) m. constantinus clade, two (i.e., Sardinia/Tuscany and Lampedusa) out of the three subgroups appear as homogeneous geographical groupings.

## ﻿Introduction

The genus *Carabus* Linnaeus, 1758 (Coleoptera, Carabidae) includes about 1000 species currently classified in over 91 subgenera. This genus is widespread in the Holarctic area but nearly all species are distributed in the Palearctic region including Japan, Iceland, Canary Islands, and North Africa, with only a few (11 species) in North America (Deuve 2004).

Carabids are mostly nocturnal predators represented by numerous brachypterous (i.e., wingless) species with low dispersal power, living in restricted areas, sometimes punctiform, and with extreme specialization towards particular environments (forests, grasslands, or agricultural landscapes) and prey (snails, earthworms, or caterpillars). Such a high degree of ecological differentiation is represented by numerous (morphological) subspecific forms (Březina 1999; Deuve 2004) but, despite the number of studies conducted so far (see Mossakowski 2003 and references therein; Osawa et al. 2004; Andújar et al. 2014), the global evolutionary history of this hyper-diverse genus still remains poorly understood.

Molecular genetic studies confirmed a substantial monophyly of the morphological subgroups of *Carabus* (Sota and Ishikawa 2004 and references therein; Deuve et al. 2012) which are subdivided into clades that diverged around 10 Mya (6.6–14.8). However, many issues on this topic remain unsolved, such as the correct dating of the speciation events using the molecular clock (Prüser and Mossakowski 1998; Andújar et al. 2012a, 2012b). In fact, following Andújar et al. (2012b), dates obtained either for the origin of the genus or for the split of different subgenera are in line with the hypothesis suggested by Deuve et al. (2012), whereas a recent study gives the origin of *Carabus* in the Eocene (Opgenoorth et al. 2021).

Within the large Carabidae family, speciation processes are probably due to geological and paleo-ecological events, and, for the Euro-Mediterranean area, they can be explained by the Eurasian forest fragmentation consequent to the Miocene climatic changes and subsequent Plio-Pleistocene climatic events (see also Prüser and Mossakowski 1998; Turin et al. 2003; Deuve et al. 2012). Particularly, during the Messinian salinity crisis (5.9‒5.3 Mya), severe environmental changes occurred in the Mediterranean region leading to the reduction of tropical forests and to more xeric (hot and dry) habitats. Species which were adapted to tropical environments became extinct (Deuve 1998) and taxa (including *Carabus*) which resulted more suited to the new climatic conditions evolved and prevailed. More specifically, the colonization of mid-Mediterranean areas is testified by the dispersion of several taxa such as the subgenus Macrothorax Desmarest, 1850 (Prüser and Mossakowski 1998), the Corso-Sardinian C. (Eurycarabus) genei Gené, 1839, C. (Macrothorax) planatus Chaudoir, 1843 (today endemic of montane forests of northern Sicily), and a number of subspecies of C. (Macrothorax) rugosus Fabricius, 1792 and C. (Rhabdotocarabus) melancholicus Fabricius, 1798 (Turin et al. 2003).

The subgenus Macrothorax was described by Desmarest (1850) and includes a group of species morphologically and geographically well-isolated in Western Mediterranean. This subgenus is considered a Tyrrhenian element, pre-Quaternary, whose diffusion and speciation are correlated with the Messinian salinity crisis and with the Plio-Pleistocene events (Jannel 1941; Antoine 1955; Darnaud et al. 1981; Casale et al. 1982; La Greca 1984; Vigna Taglianti et al. 1993; Vigna Taglianti 1998; Turin et al. 2003). *Macrothorax* comprises also populations that seem to have originated in more recent times or, likely, from passive transport (see Casale et al. 1989; Turin et al. 2003). The larva is of the rostilabrous type, which brings this subgenus closer to the groups of more oriental origin.

**Figure 1.**
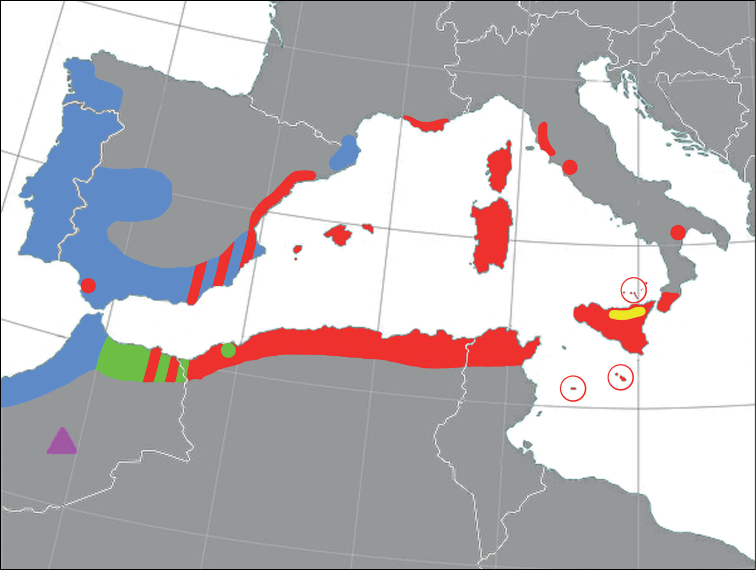
Distribution map of Carabus (Macrothorax) species in Mid Mediterranean areas. Red: C. (M.) morbillosus; yellow: C. (M.) planatus; blue: C. (M.) rugosus; green: C. (M.) aumontii; Purple triangle: C. (M.) meurguesianus.

Currently (see Löbl and Löbl 2017) the species listed in the subgenus Macrothorax are:

C. (M.) morbillosus Fabricius, 1792 (S-France, S-Spain, N-Morocco, N-Algeria, N-Tunisia, Corse, Sardinia, Tyrrhenian central Italy, Southern Calabria, Sicily, Sicilian islands and Malta);

C. (M.) rugosus Fabricius, 1792 (S-Spain, Portugal, N-Morocco);

C. (M.) aumontii Lucas, 1849 (NE-Morocco, NW-Algeria), type species;

C. (M.) planatus Chaudoir, 1843 (Sicily);

C. (M.) meurguesianus Ledoux, 1990 (Morocco) (Fig. 1).

These species live in varied habitats. Carabus (M.) morbillosus and C. (M.) aumontii mostly occur at low and medium altitudes in dense Mediterranean shrubland or palm forest soils with sufficient vegetation coverage, but also in areas with sparse vegetation or stony ground. Carabus (M.) rugosus and C. (M.) planatus are present in mountain and woodland environments.

With the exception of C. (M.) planatus, subspecies limited to well-defined geographic areas are ascribed to each of these species.

Considering only C. (M.) morbillosus, these subspecies include:

Carabus (M.) m. morbillosus Fabricius, 1792, locus typicus: “Mauretania” (Fabricius 1792).

Carabus (M.) m. alternans Palliardi, 1825, locus typicus: Sicilia (Palliardi 1825).

Carabus (M.) m. macilentus Lapouge, 1899, “sud de l’Espagne” (Lapouge 1899).

Carabus (M.) m. cychrisans Lapouge, 1899, NW-Algeria: Oran env. ? Maghnia (Lapouge 1899).

Carabus (M.) m. galloprovincialis Lapouge, 1910, locus typicus: Le Muy, Var, France (Lapouge 1910; Jannel 1941).

Carabus (M.) m. constantinus Lapouge, 1899, locus typicus: “Constantine [Algeria]” (Lapouge 1899).

Carabus (M.) m. bruttianus Born, 1906, locus typicus: “Calabria” (Born 1906).

Carabus (M.) m. arborensis Krausse, 1908, locus typicus: “Asuni [Sardinia, Italy]” (Krausse 1908).

Carabus (M.) m. corsicanus Lapouge, 1913, locus typicus: “Corsica” (Lapouge 1913).

Carabus (M.) m. lampedusae Born, 1925, locus typicus: “Lampedusa” (Born 1925).

Carabus (M.) m. cheminorum Deuve, 1988.

Many of these taxa are not accepted or have had different interpretations (Casale et al. 1982; Vigna Taglianti 1995; Březina 1999; Vigna Taglianti et al. 2002; Löbl and Smetana 2003; Turin et al. 2003; Deuve 2004; Vigna Taglianti 2009; Cavazzuti and Ghiretti 2020). The latest checklist of the Italian fauna reports C. (M.) m. morbillosus in Tuscany, Sardinia, and Sicily, C. (M.) m. alternans in Basilicata, Calabria, and Sicily, and C. (M.) planatus in Sicily (Casale et al. 2021).

The taxonomy and morphological characteristics of the main *morbillosus* subspecies accepted so far are briefly reported below.

Carabus (M.) m. morbillosus: N-Algeria SE-France (but the French populations were probably introduced from Kabylia: subsp. cheminorum). Pronotum widened anteriorly, sides arcuate, slightly narrowed anteriorly side, elytra elongate-ovate, subconvex, elytral sculpture with primary intervals catenulate, convex, 4^th^ secondary interval evident, complete, tertiary intervals reduced, broken in rows of granulations, dorsal surface often darker and more polychromous, in some populations green to blue-violet.

Carabus (M.) m. macilentus: SE-Spain (Murcia, Catalonia), Algeciras (Cádiz), Balearic Islands. Pronotum narrowed anteriorly, the sides slightly arched, very short tertiary intervals, depressed elytra, cupreous dorsal surface normally dark, or greenish, the disc of the pronotum often blackish.

Carabus (M.) m. constantinus: NE-Algeria, Tunisia, Italy (Tuscany, Lazio: probably introduced), Sardinia, Corse, Lampedusa, SE-France (introduced probably from Corse). Elytra more convex; intervals less convex, 4^th^ secondary interval fully reduced, tertiary intervals granulated but more evident than in the typical form, dorsal surface more constantly metallic bronze to reddish-cupreous.

Carabus (M.) m. alternans: Sicily, Calabria (Aspromonte), Basilicata, Malta. This population is differentiated from the other populations by a large shiny pronotum flattened posteriorly, with maximum width at middle and constricted forward; primary intervals elongated and slightly salient, secondary ribs depressed, tertiary intervals less raised than secondary ones, 1^st^ elytral interstria deeply punctured with points sometimes juxtaposed; apex of aedeagus relatively short and wide, elytra elongate, rounded and dilated in the rear third, elytra apex short and sightly sinuate at sides.

A few years ago, Rapuzzi and Sparacio (2015) proposed the validity of C. (M.) m. lampedusae (Lampedusa) and C. (M.) m. bruttianus (S-Calabria, NE-Sicily: Messina and surrounding areas, Aeolian Islands).

Carabus (M.) m. lampedusae is similar to C. (M.) m. constantinus but shows a large and convex body shape and is less bright in color. Dark pronotum with basal sulci large and deep, side sinuate before hind angle, primary intervals wider, 1^st^ elytral interstria with points on the surface, well separated from each other.

Carabus (M.) m. bruttianus is similar to C. (M.) m. alternans but is smaller and convex on elytron apex, less shiny, pronotum narrower and slightly rounded forward with maximum width in the fore half, elytra evidently shorter and oval, primary intervals in granules shorter and less raised, elytron apex stretched and clearly sinuate at side.

Likewise, Müller and Mifsud (2017) described C. (M.) m. gozomaltensis Müller & Mifsud, 2017 from Malta and Gozo Islands. These Maltese populations appear to be characterized by a smaller size and a darker coloration of the surface than Sicilian specimens, and a particular male genitalia structure.

To date interpretation of C. (M.) morbillosus subspecies remains elusive. To contribute to this problem, we used both a morphometric analysis of four morphological characters (i.e., elytra length, elytra width, pronotum length, and pronotum width), and a genetic analysis of a fragment of the cytochrome oxidase subunit I (COI) gene to determine additional information on the basal relationships among representative populations of C. (M.) morbillosus in mid-Mediterranean areas. Our focus was on populations inhabiting central mainland Italy, Sardinia, Sicily, circum-Sicilian islands, Malta, Spain, the Balearic Islands, and Tunisia.

## ﻿Materials and methods

### ﻿Materials

A total of 128 Carabus (M.) morbillosus male specimens were studied in the morphometric analysis. Samples were collected in Italy (Lampedusa, Sardinia, Calabria, Sicily, including four locations throughout the island, plus Messina province, which is interesting for its biogeographical connections with Calabria), Tunisia, and the Balearic Islands.

### ﻿Morphometric analysis

For each specimen four characters were measured: length of elytra (**EL**), width of elytra (**EW**), length of pronotum (**PL**), width of pronotum (**PW**).

Morphometric characters were used in an exploratory cluster analysis (complete linkage, Euclidean distance) to determine if the combinations of biometric characters allow to delimit groups concordant with the subspecies. Afterwards, a discriminant analysis was performed to assess the usefulness of the recorded variables to identify groups. A principal component (**PC**) analysis was then performed using the same four morphometric factors. Since one character (PL) was not available for one specimen from Sardinia, 127 male specimens were used for the analyses. Moreover, analysis of mean differences of morphometric characters among groups was then performed with ANOVA, after data normalization by means of a Box-Cox transformation. All analyses were concluded with Tukey *post hoc* tests to compare the groups for each character (*p* < 0.05). Minitab software has been used throughout for all statistical analyses.

### ﻿Molecular analysis

**Figure 2.**
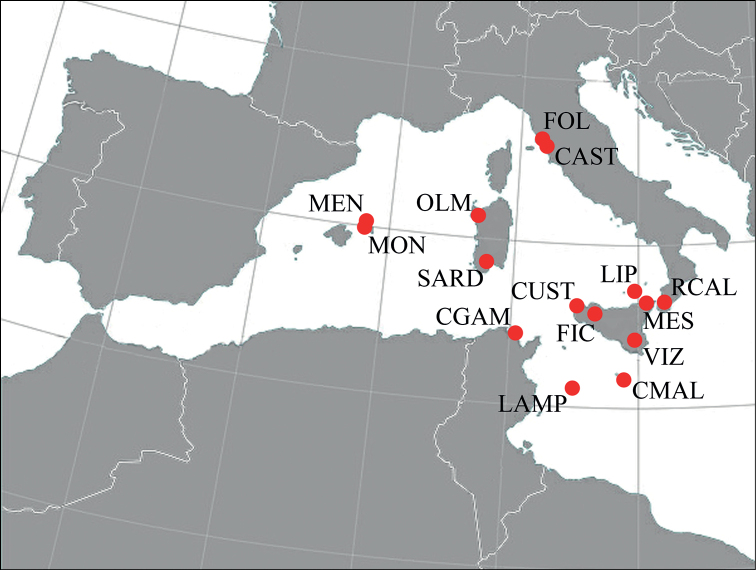
Collection sites of C. (M.) morbillosus specimens employed for molecular analyses are reported as red dots. Details and locality labels are described in the text.

Molecular analysis was performed on 38 specimens of C. (M.) morbillosus from several localities: Malta [(collection site not available, (CMAL)]; Vizzini [Italy: Sicily (VIZ)]; Custonaci [Italy: Sicily (CUST)]; Corleone, Ficuzza [Italy: Sicily (FIC)]; Messina [Italy: Sicily (MES); Reggio Calabria [Italy: S-Calabria (RCAL)]; Lipari [Italy: Aeolian Islands (LIP)]; Olmedo Prepalzos [Italy: N-Sardinia (OLM)]; Capoterra and Is Cannoneris [Italy: S-Sardinia (SARD)]; Follonica [Italy: Tuscany (FOL)]; Castiglione della Pescaia [Italy: Tuscany (CAST)]; Lampedusa [Italy: Sicily, Pelagie Islands, (LAMP)]; Cap Gammarth (Tunisia (CGAM)]; Ses Mongetes, Citadella de Menorca [Spain: Balearic Islands, Menorca (MEN)]; and Lloc de Monges, [Spain: Balearic Islands, Menorca (MON)] (Fig. 2).

Samples were stored at −20 °C in test tubes. Total genomic DNA was isolated from a small piece of tissue taken from the ethanol-preserved specimens. The extractions were carried out using the Wizard Genomic DNA Purification Kit (Promega). All the DNA extractions were kept at 4 °C for short-time use. Undiluted or different dilutions (1:10–1:50, based on the DNA concentration) of each DNA extraction were used as templates for PCR amplification of a portion of the cytochrome oxidase subunit I (mt-COI) gene.

COI amplicons were obtained by the universal internal primers LCO1490 and HCO2198 as in Folmer et al. (1994) by the following PCR protocol: 95 °C for 5 min; 95 °C for 1 min, 50 °C for 1 min, 72 °C for 1 min (35 cycles); 72 °C for 10 min. To remove primers and unincorporated nucleotides, the amplified products were purified by the Wizard SV gel and PCR Clean-up Kit (Promega). Sequencing of the purified PCR products was carried out using automated DNA sequencers at Eurofins MWG Operon (Germany). Sequence chromatograms of each amplified fragment were browsed visually. Sequences were visualized with BioEdit Sequence Alignment Editor 7 (Hall 1999), aligned with the ClustalW option included in this software and double checked by eye.

All sequences generated in the present study were deposited in NCBI GenBank (OM681023–OM681060).

Phylogenetic analyses were conducted in BEAST 1.6.1 (Drummond and Rambaut 2007) with 10×10^6^ generations and 10% burnin. The best-fit evolution model of nucleotide substitution resulted in HKY+G (gamma = 0.128) with empirical base composition; the Yule Process tree prior for mitochondrial data with piecewise linear population size model was applied with a UPGMA-generated tree as the starting point. Trees were combined to produce an ultrametric consensus tree using TreeAnnotator 1.6.1. Support for nodes is expressed as posterior probabilities.

In addition, homologous sequences (retrieved from GenBank) of *Carabusrugosus* (JQ689882, JQ689892), *C.morbillosusalternans* (JQ646591), *C.morbillosus* (JQ689896-JQ689898, JQ689883, JX279622), *C.planatus* (JQ646589), *Calosomasycophanta* (JQ693413), and *Cychrussemigranosus* (JQ689876) were included. *Campalitaauropunctatum* (JQ689899) was used as outgroup (OG).

## ﻿Results

### ﻿Morphometric data

**Figure 3.**
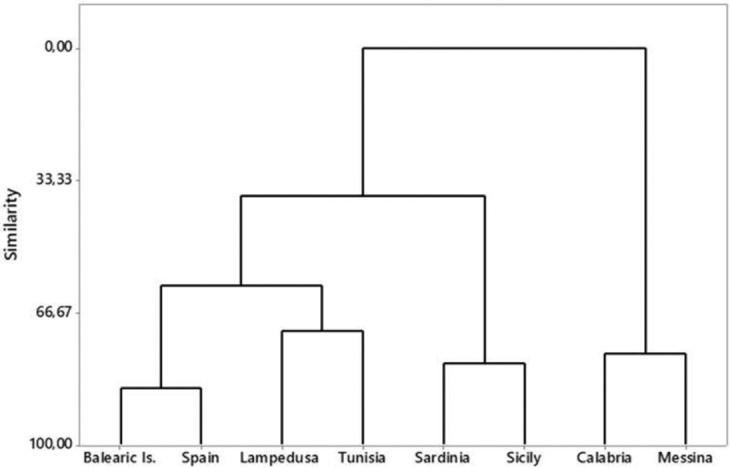
Similarity tree obtained from the cluster analysis based on the means of the four morphometric parameters. Morphological characters measured were as follows: elytra length, elytra width, pronotum length, pronotum width.

The dendrogram obtained from the cluster analysis (Fig. 3) highlights the localities which clearly group together. These are: (i) Messina/Calabria; (ii) Spain/Balearic Islands; and (iii) Tunisia/Lampedusa, corresponding respectively to the subspecies C. (M.) m. bruttianus, C. (M.) m. macilentus, and C. (M.) m. constantinus. The population from Sardinia, based on the morphometric characters used, would seem closer to the Sicilian population [C. (M.) m. alternans], although this requires further investigation, which certainly needs to consider other additional morphological characters. Moreover, despite the morphometric similarity between the populations from Tunisia and Lampedusa, we prefer to consider these two populations as separate groups in the following biometric analyses, thus analyzing separately all the populations which have been alternatively included in the C. (M.) m. constantinus group (Sardinia, Tunisia, and Lampedusa).

The results of the discriminant analysis, conducted on the groups from Sicily, Sardinia, Tunisia, Lampedusa, Messina/Calabria, and Spain/Balearic Islands, reveals that the proportion of correct attribution is 0.646 (Table 1). A higher correct classification of samples was found for Messina/Calabria and Spain/Balearic Islands (0.85 and 0.78, respectively), while the highest misclassification was found for Sardinia, with less than 50% correctly classified. For all the other populations, the proportion of correct classification ranged between 0.54 (Tunisia) and 0.61 (Lampedusa), thus confirming the need of a more comprehensive approach for a better characterization. The linear discriminant function shows that PL was the most relevant parameter in the group attribution, followed by EL and PW, while EW resulted the less discriminant parameter.

**Table 1.** Classification of Carabus (Macrothorax) morbillosus male specimens of the different groups determined by the discriminant analysis performed on all parameters. Coefficients of the discriminant function show the impact of each parameter in the correct attribution to the four groups.

	Lampedusa	Messina/Calabria	Sardinia	Sicily	Spain/Balearic Is.	Tunisia
Lampedusa	14	0	3	1	0	4
Messina/Calabria	0	28	2	4	3	0
Sardinia	1	1	8	2	0	1
Sicily	1	3	5	11	0	0
Spain/Balearic Is.	2	1	2	2	14	1
Tunisia	5	0	0	0	1	7
Total *N*	23	33	20	20	18	13
*N* correct	14	28	8	11	14	7
Proportion	0.61	0.85	0.4	0.55	0.78	0.54
*N* = 127		*N* correct = 82		Proportion correct = 0.646		
Linear Discriminant Function for groups						
	Lampedusa	Messina/Calabria	Sardinia	Sicily	Spain/Balearic Is.	Tunisia
Constant	−345.05	−274.38	−298.79	−303.67	−332.05	−366.62
Elytra width	−0.20	−0.16	−0.11	−0.08	−0.21	−0.20
Elytra length	2.20	1.73	1.84	1.86	2.01	2.27
Pronotum width	1.91	1.57	1.88	1.63	1.64	1.78
Pronotum length	2.24	2.84	2.43	2.71	2.97	2.56

Results of the statistical analysis for the examined morphometric parameters are reported in Table 2. Significant differences among groups were found for all considered parameters. The group of specimens from Calabria/Messina shows significantly lower values in three out of four parameters (EW, EL, and PW), confirming that C. (M.) m. bruttianus is clearly smaller than the other three groups. Sicily and Sardinia differed from Lampedusa and Tunisia for EL and PW. As expected, no significant differences were found between Sicily and Sardinia and between Tunisia and Lampedusa for all morphometric characters.

**Table 2.** Biometrics (mean ± S.E.) of males of the different Carabus (Macrothorax) morbillosus groups. Different letters within the column indicate significant differences among group means (One-way ANOVA performed after Box-Cox transformation of data: EW*F*_5,122_ = 11.52; EL*F*_5,122_ = 46.58; PW*F*_5,122_ = 22.94; PL*F*_5,121_ = 10.84 followed by Tukey post-hoc test, *p* < 0.05).

Groups	No.	EW ± SE (min-max)	EL ± SE (min-max)	PW ± SE (min-max)	PL ± SE (min-max)
Calabria/Messina	33	103.94 ± 2.36 c	166.45 ± 1.14 d	71.55 ± 0.61 d	58.36 ± 0.40 b
		(86–150)	(151–178)	(65–81)	(55–63)
Spain/Balearic Is.	18	108.28 ± 0.94 bc	184.72 ± 1.79 b	77.72 ± 1–16 bc	63.33 ± 0.63 a
		(100–114)	(170–200)	(70–88)	(59–68)
Tunisia	13	116.62 ± 1.99 ab	197.31 ± 2.97 a	82.38 ± 1.06 a	63.85 ± 0.96 a
		(105–130)	(180–220)	(77–90)	(59–70)
Lampedusa	23	113.43 ± 1.51 ab	191.43 ± 1.70 ab	81.61 ± 1.09 ab	60.65 ± 0.96 b
		(100–133)	(180–210)	(74–90)	(54–70)
Sardinia	21 (20 for PL)	124.71 ± 5.28 ab	175.57 ± 1.79 c	77.60 ± 0.70 c	58.57 ± 0.72 b
		(99–170)	(153–190)	(70–82)	(53–64)
Sicily	20	131.00 ± 5.48 a	177.25 ± 1.44 c	76.15 ± 0.85 c	60.45 ± 0.34 ab
		(98–180)	(166–192)	(70–82)	(58–64)

**Figure 4.**
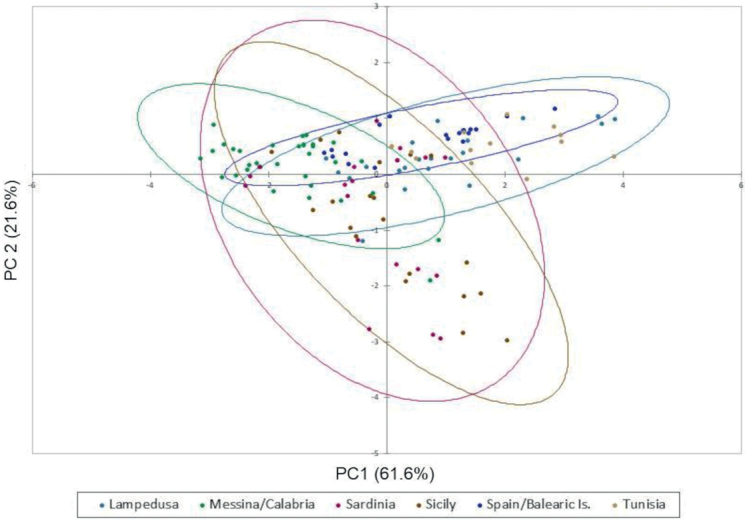
The principal component analysis applied to morphometric characters explained 83.2% of all variance. The first principal component (61.6%) is related to PL, PW and EL, whereas the second one (21.6%) is related to EW.

The PC analysis indicates that the four morphometric characters explained 83.2% of all variance, mainly related to PL, PW, and EL (PC1, 61.6%) (Fig. 4). Despite the overlapping of the different groups, the PC2 (mainly related to EW) seems to have a more relevant role in the two populations from Sicily and Sardinia compared to Lampedusa, Tunisia, and Spain/Balearic Islands.

### ﻿Molecular data

**Figure 5.**
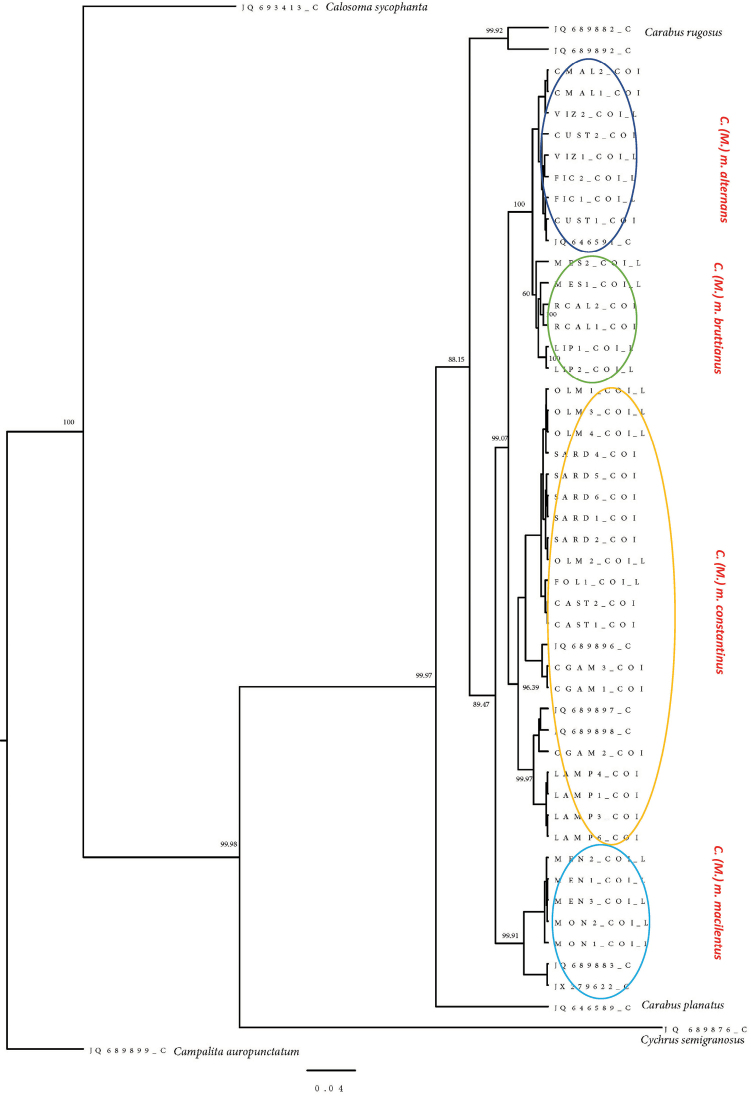
50% majority rule Bayesian tree inferred from dataset including partial sequences of the mitochondrial COI genes available in the present paper along with homologous sequences retrieved from GenBank (see text for details). Nucleotide substitution model: HKY + G (gamma = 0.128). Numbers above branches represent Bayesian posterior probabilities. Scale bar represents units of length in expected substitutions per site.

As shown in Fig. 5, the Bayesian analysis using COI partial sequences reveals several clearly distinct clusters. It is possible to distinguish the different genera included in the study, in particular, the genus *Calosoma* (represented by *Calosomasycophanta*), the genus *Carabus* (represented by the species C. (M.) rugosus, C. (M.) morbillosus sspp., and C. (M.) planatus), the genus *Cychrus* (represented by *C.semigranosus*), and the genus *Campalita* (represented by *C.auropuctatum*, which in our analysis was chosen as the outgroup). Relationships between these genera are supported by a clear tree topology with high posterior probability values at the main nodes. Among *Carabus* species, posterior probability values are very high, and affinity relationships can be easily deduced from the tree topology. Carabus (M.) rugosus is sister of C. (M.) morbillosus, with C. (M.) planatus slightly more distant and sister of the clade C. (M.) rugosus/C. (M.) morbillosus. At the subspecific level, it is possible to clearly distinguish within C. (M.) morbillosus four clusters: the first contains the sequences of the specimens from Malta and Sicily together with a sequence retrieved from GenBank database (JQ646591) of a specimen sampled in “Italy” and reported as *C.morbillosusalternans*. According to our interpretation, this cluster contains specimens that can be ascribed with reasonable certainty to the subspecies C. (M.) m. alternans. The second group includes specimens from Messina (NE-Sicily), Reggio Calabria (S-Calabria), and Lipari (Aeolian Islands) and, in our opinion, these specimens belong to the subspecies C. (M.) m. bruttianus. The third group includes specimens from Sardinia, Tuscany, Tunisia, and Lampedusa plus three sequences (from Tunisia) from GenBank (JQ689896‒JQ689898). This group, which is the biggest one, represents the subspecies C. (M.) m. constantinus. Finally, the fourth group includes specimens from the Balearics plus two sequences (JQ689883 and JX279622) from Spain reported as *C.morbillosus.* This group, in our interpretation, represents the subspecies C. (M.) m. macilentus. As regards the distances expressed in *p* distance (i.e., number of nucleotide substitutions), the subspecies alternans is 0.038 far from *constantinus*, and 0.045 from *macilentus*. A very small distance (0.014) separates *alternans* from *bruttianus*.

## ﻿Discussion

The results of the morphometric and molecular analyses in this study show a significant agreement between hypothesized relationships of taxa. Combining information from the similarity tree and the phylogenetic tree, the validity of the subspecies alternans, *bruttianus*, *constantinus*, and *macilentus* is supported.

The subspecies bruttianus is only separated by a small genetic distance (ca 1%) from *alternans*, but the subspecific rank is supported by the tree topology. In addition, a comparison between the Calabria/Messina clustering obtained with morphometric analysis and the MES+RCAL+LIP cluster in the Bayesian tree clearly supports the validity of the subspecies bruttianus, as proposed by Rapuzzi and Sparacio (2015). As shown in Fig. 5, the posterior probability supporting the cluster is 60%. On closer examination, the RCAL and LIP sequences are very homogeneous (100%, each), so the overall posterior probability value drops to 60% due to the greater heterogeneity observed in the sequences of the Messina specimens. Given that it is probably necessary to analyze many more beetles from the hypothesized distribution area of *bruttianus*, these results may be explained by Messina specimens having undergone more rapid molecular change than morphological change. This could explain the difference in COI despite being rather morphologically similar to continental ones.

Within the large clade *constantinus*, out of three subgroups, two homogeneous geographic groupings were found in Sardinia (including also Tuscany) and Lampedusa, whereas the third one (Tunisia) appears to be more heterogeneous. Of the Sardinian specimens, all individuals cluster within the *constantinus* group, while the morphometric analysis shows them to be closer to *alternans*.

The molecular similarity between Sardinian and Tuscan populations is in agreement with their morphological similarity which, depending on different hypotheses, is considered the result of ancient passive transport (i.e., by anthropogenic transport, perhaps by the Phoenicians; Casale et al. 1989; Turin et al. 2003) or native (Vigna Taglianti 1998). If we wanted to distinguish at the subspecific level the populations of Sardinia and central Italy (Tuscany), *arborensis* could be used. However, we are fully aware that the present data do not allow us to draw any definitive conclusions, which is worth exploring in a future study.

Although Lampedusa specimens are all included in a homogeneous geographical subgroup, the subspecific rank is only partially supported by the tree topology using COI data. However, such an outcome does not necessarily affect the validity of the subspecies which was diagnosed morphologically. Combining molecular with morphological monophylies, a subgenus is supported, but more in-depth study is needed by analyzing more morphological characters, more beetle specimens, and more genes (at least one nuclear) to obtain a clearer insight on the evolutionary paths followed by *morbillosus* in Italy and Tunisia. Of course, this larger study must also include beetles from Algeria, northern Morocco, and southeastern Spain.

In conclusion, our results provide new evidence supporting the validity of the C. (M.) morbillosus subspecies, C. (M.) m. alternans, C. (M.) m. bruttianus, C. (M.) m. constantinus, and C. (M.) m. macilentus, and we can redefine their distribution in mid-Mediterranean areas.

One latter consideration refers to C. (M.) planatus which was shown in the phylogenetic tree as the most distant *Macrothorax* species analysed. It is an endemic species that lives exclusively in the Nebrodi and Madonie woods of Sicily (Magistretti 1965; Bruno 1968; Rapuzzi 1992; Sparacio 1995; Busato and Casale 2004), at higher altitudes; externally, it looks like C. (M.) rugosus of Spain and Morocco. Darnaud et al. (1981) reported it as the most primitive species of the subgenus Macrothorax, in agreement with other authors (Prüser and Mossakowski 1998; Turin et al. 2003) who considered C. (M.) planatus as one of the most ancient species of the Mediterranean *Macrothorax*. This species was confused with C. (M.) morbillosus for many years (see Casale et al. 1982), despite that many authors, including Chaudoir (1843), noted that C. (M.) morbillosus and C. (M.) planatus are not the same species (see also Ragusa 1871, 1883, 1908, 1921; Vitale 1912), and this is worth further study.
